# Functional imaging and circulating biomarkers of response to regorafenib in treatment-refractory metastatic colorectal cancer patients in a prospective phase II study

**DOI:** 10.1136/gutjnl-2017-314178

**Published:** 2017-08-08

**Authors:** Khurum Khan, Mihaela Rata, David Cunningham, Dow-Mu Koh, Nina Tunariu, Jens C Hahne, George Vlachogiannis, Somaieh Hedayat, Silvia Marchetti, Andrea Lampis, Mahnaz Darvish Damavandi, Hazel Lote, Isma Rana, Anja Williams, Suzanne A Eccles, Elisa Fontana, David Collins, Zakaria Eltahir, Sheela Rao, David Watkins, Naureen Starling, Jan Thomas, Eleftheria Kalaitzaki, Nicos Fotiadis, Ruwaida Begum, Maria Bali, Massimo Rugge, Eleanor Temple, Matteo Fassan, Ian Chau, Chiara Braconi, Nicola Valeri

**Affiliations:** 1 Department of Medicine, The Royal Marsden NHS Trust, London and Sutton, UK; 2 Division of Molecular Pathology, The Institute of Cancer Research, London and Sutton, UK; 3 Division of Radiotherapy and Imaging, Cancer Research UK Imaging Centre, The Institute of Cancer Research and Royal Marsden Hospital, London, UK; 4 Division of Cancer Therapeutics, The Institute of Cancer Research, London and Sutton, UK; 5 Department of Statistics, The Royal Marsden NHS Trust, London and Sutton, UK; 6 Department of Medicine (DIMED) and Surgical Pathology, University of Padua, Padua, Italy

**Keywords:** regorafenib, anti-angiogenic treatment, cancer therapeutics, DCE-MRI, liquid biopsy, colorectal cancer

## Abstract

**Objective:**

Regorafenib demonstrated efficacy in patients with metastatic colorectal cancer (mCRC). Lack of predictive biomarkers, potential toxicities and cost-effectiveness concerns highlight the unmet need for better patient selection.

**Design:**

Patients with *RAS* mutant mCRC with biopsiable metastases were enrolled in this phase II trial. Dynamic contrast-enhanced (DCE) MRI was acquired pretreatment and at day 15 post-treatment. Median values of volume transfer constant (K^trans^), enhancing fraction (EF) and their product KEF (summarised median values of K^trans^× EF) were generated. Circulating tumour (ct) DNA was collected monthly until progressive disease and tested for clonal *RAS* mutations by digital-droplet PCR. Tumour vasculature (CD-31) was scored by immunohistochemistry on 70 sequential tissue biopsies.

**Results:**

Twenty-seven patients with paired DCE-MRI scans were analysed. Median KEF decrease was 58.2%. Of the 23 patients with outcome data, >70% drop in KEF (6/23) was associated with higher disease control rate (p=0.048) measured by RECIST V. 1.1 at 2 months, improved progression-free survival (PFS) (HR 0.16 (95% CI 0.04 to 0.72), p=0.02), 4-month PFS (66.7% vs 23.5%) and overall survival (OS) (HR 0.08 (95% CI 0.01 to 0.63), p=0.02). KEF drop correlated with CD-31 reduction in sequential tissue biopsies (p=0.04). *RAS* mutant clones decay in ctDNA after 8 weeks of treatment was associated with better PFS (HR 0.21 (95% CI 0.06 to 0.71), p=0.01) and OS (HR 0.28 (95% CI 0.07–1.04), p=0.06).

**Conclusions:**

Combining DCE-MRI and ctDNA predicts duration of anti-angiogenic response to regorafenib and may improve patient management with potential health/economic implications.

**Significance of the Study**

**What is already known on this subject?**

- Regorafenib is approved as third-line therapy for patients with refractory colorectal cancer; however, its use in the clinic has been restricted due to modest clinical benefit in unselected patients.
- Published preclinical studies suggested that anti-angiogenic activity of regorafenib is the main predeterminant of its efficacy but no clinical studies have validated these findings.
- Retrospective analysis of prospective clinical trials failed to identify biomarkers of response to regorafenib that might be implemented in clinical practice.

**What are the new findings?**

- Regorafenib showed significant activity in patients with marked early anti-angiogenic response, resulting in a longer disease control, better progression-free survival and overall survival.
- Early (day 15 post-treatment) dynamic contrast enhanced (DCE)-MRI predicts response and long-term outcome during regorafenib treatment.
- Sequential analysis of tissue biopsies confirmed that reduction in tumour vasculature as the mechanism underpinning the observed radiological findings.
- Persistent regorafenib-induced anti-angiogenic effect translates into a reduction in circulating tumour (ct) DNA and this might be incorporated into the clinical algorithm for patients’ management.

**Implications on clinical practice**

Implementing the use of DCE-MRI and ctDNA analysis as early biomarkers of response to regorafenib might improve patient selection with clear health/economic implications for patients, health systems and society.

## Introduction

Colorectal cancer (CRC) remains a major health burden with significant morbidity and mortality despite recent improvements in its management owing to better screening and therapeutic options.^1^ CRC is known to be a biologically heterogeneous disease characterised by the activation of several angiogenic and oncogenic pathways.^2^ Regorafenib, a multikinase inhibitor with known anti-angiogenic, antistromal and anti-oncogenic activities,^3^ has demonstrated single agent efficacy in patients with treatment refractory metastatic CRC (mCRC).^4 5^ The use of regorafenib in the clinic is however hampered by the modest efficacy in an unselected patient population, a significant side effect profile and the high drug costs. Consequently, identification of predictive biomarkers of response and resistance to regorafenib is critical for treatment stratification and appropriate patient selection such that treatment benefits could be optimised.

Several efforts are currently ongoing to define gene signatures^6^ and biomarkers of response to anti-angiogenic drug in CRC and other cancers^7^; however, validation of these biomarkers will only determine their use in clinical practice. While recent studies using tissue^8^ and plasma^9 10^ have attempted to elucidate the response and resistance mechanisms to regorafenib, the search for a clinically useful biomarker has been largely unsuccessful. A growing body of preclinical evidence suggests strong anti-angiogenic and pro-apoptotic effects of regorafenib^11–14^ with clinical data demonstrating that drug activity is independent of the tumour’s mutational status.^8^ These findings strengthen the hypothesis that additional mechanisms other than oncogenic blockade are responsible for the antitumour activity of this drug. Dynamic contrast-enhanced (DCE) MRI may have a useful role in evaluating tumour vascular heterogeneity and early anti-angiogenic effects^15 16^; moreover, its parameters volume transfer constant (K^trans^), enhancing fraction (EF) and initial area under the gadolinium concentration time curve over 60 s (IAUGC_60_) have been correlated with microvessel density and in some tumours with degree of vascular endothelial growth factor (VEGF) expression.^17^ By contrast, diffusion-weighted MRI (DW-MRI) offers useful information that reflects tumour cellularity and increase in its quantitative parameter apparent diffusion coefficient (ADC) has been associated with tumour cell death and necrosis.^18 19^ At least two preclinical studies demonstrated that regorafenib was able to significantly suppress tumour vascularity when quantified by DCE- computed tomography (CT) and MRI modalities respectively in human colon carcinoma xenograft models.^14 20^

In this prospective phase II trial of patients with *RAS* mutant mCRC treated with single agent regorafenib, we hypothesised that (1) an early anti-angiogenic and antiproliferative activity of regorafenib might be detected by multiparametric DCE-MRI on day 15 of the treatment, (2) the depth of anti-angiogenic response detected by a significant drop in DCE-MRI quantitative parameters might correlate with clinical efficacy, (3) analysis of sequential tissue and liquid biopsies could be integrated into the biomarker discovery process and shed insights into mechanisms of response to regorafenib.

## Material and methods

### Clinical trial design

**Figure 1:**
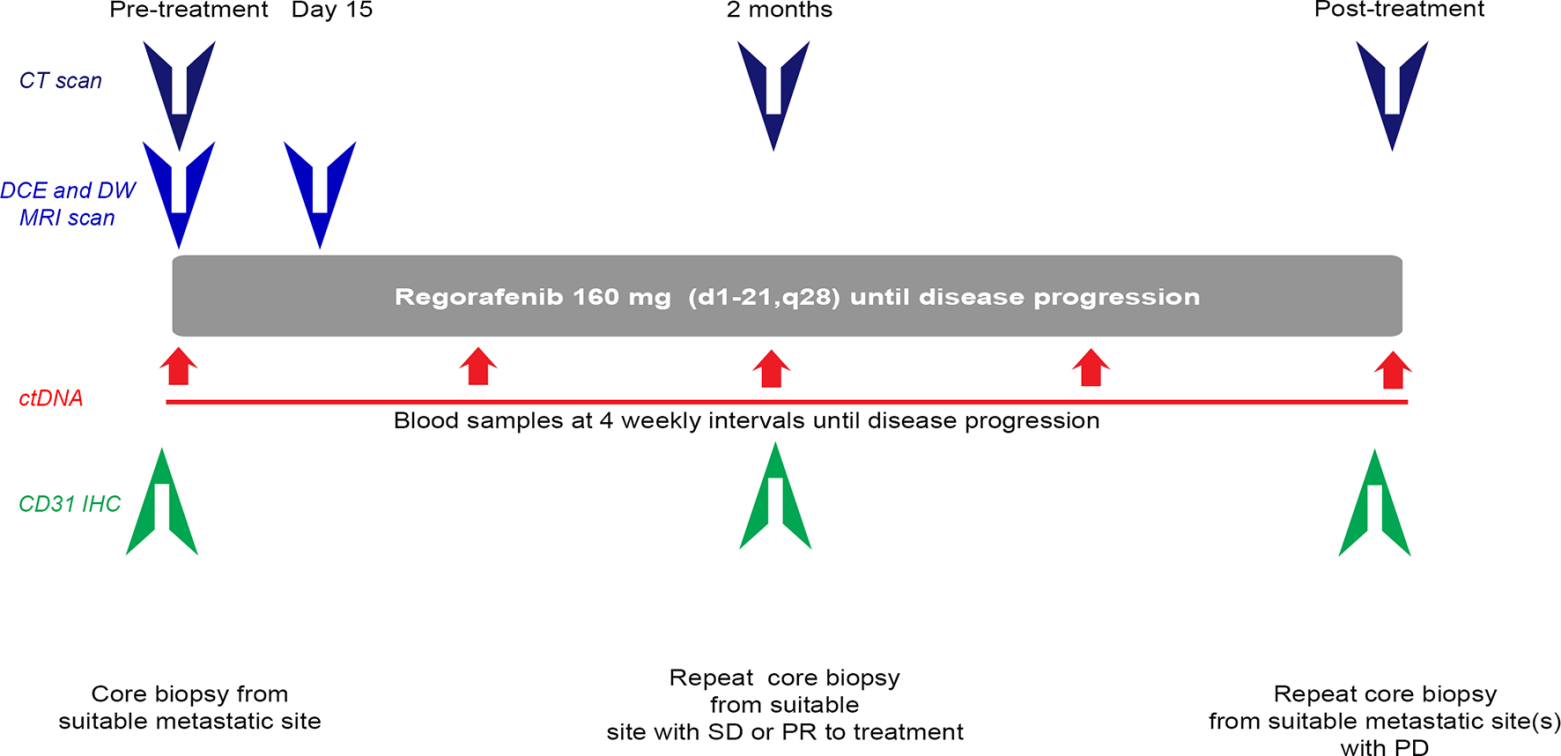
PROSPECT-R trial design. Patients meeting all inclusion and no exclusion criteria were required to have pretreatment CT, DCE-MRI and DW-MRI scans; MRI scans were then repeated on day 15. All patients were also required to have pretreatment mandatory core biopsy, followed by a core biopsy at 2 months if they had SD or PR. Patients were monitored by CT scans every 2 months until the time of PD and if clinically feasible, they had biopsy of one or two progressing lesions from PD sites. Plasma samples were collected every 4 weeks until the time of PD. ctDNA, circulating tumour DNA; DCE, dynamic contrast enhanced; DW, diffusion weighted; MRI, magnetic resonance imaging; PD, progressive disease; PR, partial response; SD, stable disease.

*PROSPECT-R* trial (clinical trials.gov number (NCT03010722)) is a phase II, open label, non-randomised study of regorafenib in patients with *RAS* mutant, chemorefractory mCRC (figure 1). Patients who were at least 18 years old and had a WHO performance status (PS) of 0–1 were deemed eligible if all conventional treatment options including fluorouracil, irinotecan, oxaliplatin and at least one anti-VEGF drugs (later trial protocol was amended due to changes in availability of anti-VEGF agents due to funding restrictions in UK) were exhausted; they had metastatic tumour amenable to biopsy and repeat measurements with DCE-MRI. Written informed consent was obtained from all patients. The study was carried out in accordance with the Declaration of Helsinki and approved by National Institutional review boards (Medicines and Healthcare products Regulatory Agency: 15983/0249/001–0001). All participants were required to have mandatory pretreatment biopsies (six cores targeted towards the MRI identified index lesion), biopsies at 2 months (if response or stable disease by RECIST V.1.1 criteria (six cores)) and at the time of progression (6–12 cores from two suitable progressing metastatic sites). Three out of six cores were snap-frozen; one core was used to establish patient- derived organoids and two cores were formalin fixed and paraffin embedded (FFPE).

The Results section describes the number of cores used for immunohistochemistry analysis in the current study. Further genomic, transcriptomic and functional analyses are ongoing on the remaining cores. Patients with suitable metastatic disease (defined as lesions at least 2 cm in diameter) and no contraindications to MRI underwent multiparametric MRI studies including matched DCE and DW; images were acquired <7 days prior to therapy and at day 15 post-treatment. Treatment consisted of regorafenib 160 mg once daily on a schedule of 3 weeks on and 1 week off until progression or intolerable side effects. More details on inclusion and exclusion criteria and criteria for patients’ withdrawal on the study are provided in the online supplementary material.

### MRI data processing

DCE-MRI data were postprocessed using the MRI Workbench software developed at our institution.^21^ The pharmacokinetic analysis was based on the extended Kety/Tofts model in conjunction with a cosine-based arterial input function model derived from population-averaged values.^22 23^ DCE-MRI parameters including K^trans^, IAUGC_60_ and the EF were obtained for pretreatment/post-treatment datasets. K^trans^ estimates were reported for both whole tumour (K^trans^(all)) and valid voxels only (K^trans^(non-zeros), i.e. excluding all non-enhancements and non-model fits) in order to address the extended necrosis observed in the cohort. The EF was defined as percentage of the voxels that enhance above the noise floor out of all tumour voxels. A voxel was considered enhancing when its postcontrast (Dotarem, Guerbet, France) dynamic intensity signal was at least 1 standard deviation higher than the mean precontrast signal, for a period of 60 s postcontrast onset. Finally, volume change in tumour enhancement during therapy (such as new necrosis) was accounted for by reporting a composite parameter, KEF, which is the product of summarised median values of KEF= K^trans^ (non-zeros) × EF.^24^ For KEF, an receiving operating charactteristic (ROC) curve analysis was performed to establish the cut-off able to identify meaningful clinical benefit based on disease control rate (DCR), progression-free survival (PFS) and overall survival (OS).

## Digital-droplet PCR

The QX200 digital-droplet PCR (ddPCR) system (Bio-Rad, Berkeley, California, USA) was used, and all reactions were prepared using the ddPCR supermix with no dUTPTP for probes. All PCR reactions were performed as duplex PCR using the relevant digital PCR assays for the wild-type and the mutation in question. Droplets were generated using the QX200 droplet generator according to the manufacturer’s protocols. The PCR reaction was performed in a C1000 Touch Thermo Cycler (Bio-Rad) using the following protocol: 95°C for 10 min followed by 40 cycles of 94°C for 30 s and 55°C for 1 min, then 98°C for 10 min. Droplets were read in the QX200 droplet reader and analysed using the Quantasoft software V. 1.6.6.0320 (Bio-Rad). Fractional abundance (FA) was defined as follows: FA % = (N*mut/*(N*mut* + N*wt*))×100), where N*mut* is the number of mutant events and N*wt* is the number of WT events per reaction. The number of positive and negative droplets was used to calculate the concentration of the target and reference DNA sequences and their Poisson-based 95% CI. ddPCR analysis of normal control plasma DNA (from cell lines) and no DNA template controls were always included. Samples with very low positive events were repeated at least twice in independent experiments to validate the obtained results as previously described.^25^

### CD31, Ki-67 and Caspase-3 immunohistochemical staining

The immunohistochemical expression of microvascular density (CD31; clone ab28364, Abcam, Cambridge, UK; dilution 1:50), cell proliferation (Ki-67; clone ab16667, Abcam; dilution 1:100) and cell apoptosis (Cleaved Caspase-3 (Asp175) (5A1E) ab9664S, Abcam; dilution 1:100) was examined on consecutive 4 µm FFPE sections of the neoplastic cores. Reactions were performed using the automated Benchmark XT platform (Ventana Medical Systems, Basel, Switzerland). Appropriate positive and negative controls were run concurrently.

For assessment of tumour microvascular density, CD31-positive microvessels were quantified and reported as the average number in 10 random fields at ×200 magnification. Ki-67 labelling index was assessed as the average number of proliferating cells in 10 random fields at ×200 magnification. Caspase-3 evaluation was categorised as positive or negative.

#### Statistical analysis

The DCR was defined by the sum of complete responses + partial responses + stable diseases using RECIST V.1.1. PFS was measured from start of treatment to date of progression or death from any cause. OS was defined as time from start of treatment to death of any cause. Patients without an event were censored at last follow-up. Response according to KEF (K^trans^ (non-zeros) × EF) was defined as a drop of >70% from baseline while change in CD31 biomarker levels from baseline was calculated as ((8 weeks−baseline)/baseline] ×100. CD31 change from baseline was explored on a continuous scale and was also dichotomised using the median value.

Response according to KEF parameter and the dichotomised CD31 change from baseline were cross-tabulated with the RECIST measured DCR. χ^2^ or Fisher’s exact tests were employed to explore whether there is an association between them and DCR. Logistic regression was employed to produce ORs and 95% CIs. The PFS and OS rates were estimated using the Kaplan-Meier method, and survival curves were generated for each group. The log-rank test was used to compare the survival curves and a Cox proportional hazards model was fitted to obtain HRs and 95% CIs. The proportional hazards assumption was tested with the use of Schoenfeld residuals.

In our study, despite relatively small study cohort, the changes in K^trans^ and KEF values were noticeably larger (eg, >50% reduction in mean and median KEF). Based on results of the 23 analysable patients evaluated by DCE-MRI in our study, our patient sample size by post hoc analysis (based on Wilcoxon signed-rank test) demonstrated 100% power to detect this difference at a level of significance of 0.05.

Additional methods can be found in the online appendix.

## Results

### Patients’ characteristics and tissue collection

Twenty-seven treated patients (63% males) were recruited in the DCE-MRI PROSPECT-R trial, and a total of 143 cores were collected by tissue biopsies from 70 metastatic lesions for the current analysis. Right and left-sided primary cancers were equally distributed in the study population; other relevant patient characteristics are summarised in table 1.

**Table 1:** Baseline characteristics of participating patients

	n	*%*
Age, median (range)	63.7 (36.3–79.0)	
Gender		
Female	10	37
Male	17	63
Site of primary		
Rectal	7	26
Left colon	9	33
Right colon	11	41
Histology diagnosis		
Unknown	1	4
Adeno (mucinous)	4	15
Adeno (non-mucinous)	22	81
Stage diagnosis		
Stage II	5	19
Stage III	5	19
Stage IV	17	62
Radiotherapy to primary		
Yes	4	15
No	23	85
Number of lines in metastatic setting		
1	1	4
2	11	41
3	9	33
4	3	11
5	2	7
6	1	4

Fifty-four tissue cores were obtained frombaseline biopsies of 27 treated (27 lesions) patients; of the 14 patients with SD at 8 weeks, 24 tissue cores were obtained from 12 (12 lesions) patients (one patient missed the biopsy due to a hospital admission secondary to chest infection and the other developed treatment-related rectal wall perforation). A further 65 tissue cores were obtained from 23 evaluable patients (35 lesions in total; 12 patients with two progressing lesions each) with PD (three patients did not complete two cycles of treatment and one came off due to treatment-related rectal wall perforation). There was 89% concordance between target DCE-MRI and biopsied metastatic lesions (see online appendix table A1). Two FFPE cores per patient were tested at each time point. One-hundred and nine plasma samples were tested to track *RAS* mutant clones in 21 corresponding patients; patients were required to have at least one sample available at 2 months following treatment.

10.1136/gutjnl-2017-314178.supp1 Supplementary Appendix 1

### Radiological and pathological evidence of early regorafenib induced anti-angiogenic effects

A significant drop in all DCE-MRI parameters was seen after 2 weeks of treatment; median K^trans^, IAUGC_60_, EF and KEF product decreased by 27.8% (IQR 6.7–52.6), 57.7% (32.7–67.9), 35.3% (12.4–56.2) and 58.3% (28.3–76.1) (see online appendix Table A2). The ROC curve analysis performed for the KEF showed that a 69.21% reduction from baseline had 100% specificity and overall accuracy of 69.57%; for pragmatic reasons, a minimum KEF product reduction of 70% was chosen (see online appendix table A3). Matched tissue analysis revealed a strong concordance between a drop in KEF and mean vascular density of tissue, as measured by CD31 count obtained pretreatment and at 2 months in patients with tissue and MR parameter data available (p=0.04) (see online appendix table A4).

### Correlation of functional imaging data and CD31 staining with clinical parameters

**Figure 2:**
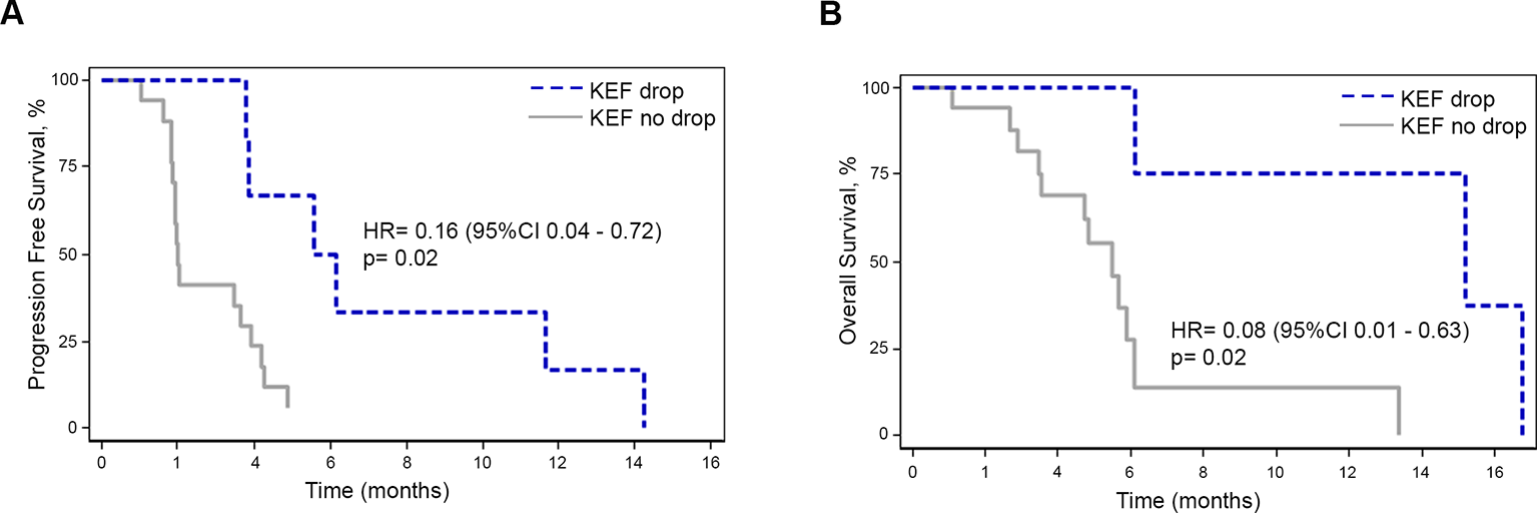
Outcome according to radiological parameters in the PROSPECT-R trial. Kaplan-Meier curves for progression-free survival (A) and overall survival (B) in patients with or without KEF drop. KEF, K^trans^× enhancing fraction.

After a median follow-up of 14.3 months ((95% CI 4.9—not evaluable (NE)), IQR 4.9—not reached), 23 patients, who had at least one cycle of regorafenib and a response assessment by CT scan at 2 months were analysable. DCR at 2 months, median PFS and median OS were 51.9%, 3.6 months (95% CI 1.9 to 4.2 months) and 5.8 months (95% CI 4.7 to 13.3 months), respectively; 77.4% (95% CI 54.0% to 89.9%), 48.0% (95% CI 24.1% to 68.5%) and 32.0% (95% CI 11.2% to 53.4%) of patients were alive at 4, 6 and 12 months, respectively. Patients with >70% drop in KEF (8/27; two patients did not undergo the 2-month scan due to treatment-related toxicities and thus were excluded from the final analysis as per the study protocol) were found to have higher DCR (6/6 vs 0/6, p=0.05) at 2 months (see online appendix table A5), better PFS (HR 0.16 (95% CI 0.04 to 0.72), p=0.02), better PFS at 4 months (66.7% vs 23.5%) and better OS (HR 0.08 (95% CI 0.01 to 0.63), p=0.02). For the group with >70% drop in KEF, 6-month and 12-month OS were 100% (95% CI NE) and 75% (95% CI 12.8% to 96.1%), respectively compared with 27.6% (7.2%–53.2%) and 13.8% (1.0%–42.5%) in the <70% drop in KEF group (figure 2A,B; see online appendix figure A1 and appendix table A6). In order to address the relative improvement in efficacy with or without KEF drop, we compared the outcomes of all the patients who achieved DCR; PFS was found to be 5.6 vs 4.2 months (HR 0.30 (95% CI 0.06 to 1.49), p=0.140) and OS was 15.2 vs 5.8 months (HR 0.11 (95% CI 0.01 to 1.06), p=0.057) in this analysis. Interestingly, when the same analysis was repeated with the cut-off chosen by ROC analysis (69.21%), PFS (HR 0.18 (95% CI 0.03 to 0.91), p=0.038) and OS (HR 0.11 (95% CI 0.01 to 1.01), p=0.051) were found to be statistically significant despite small numbers.

**Figure 3:**
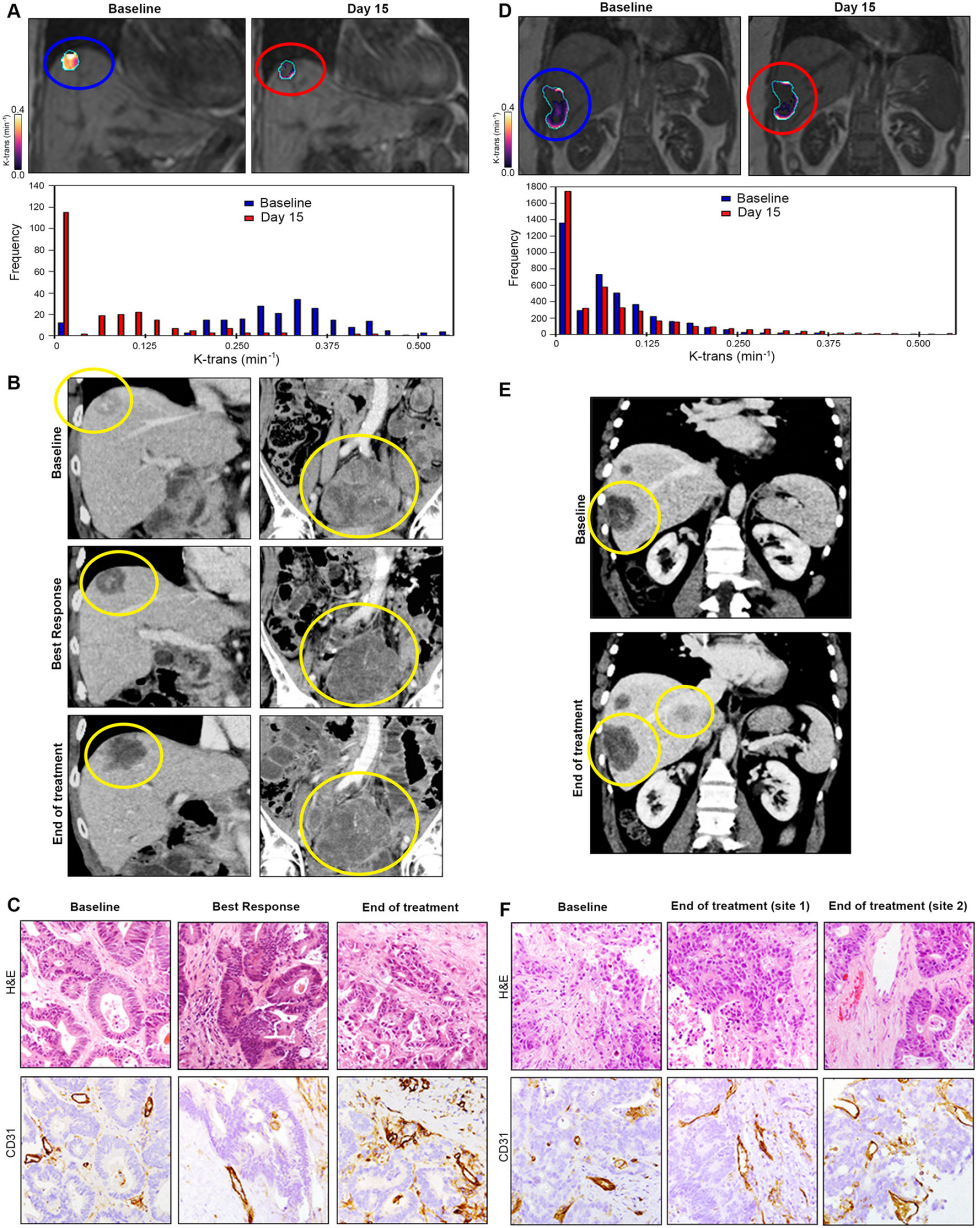
Correlation between radiological and pathological findings in the PROSPECT-R trial. Panels A–C demonstrate an example of a patient with durable disease control of 14 months, while panels D–F show example of a primary resistance patient (2 months). (A) Coronal DCE-MRI (central slice of a liver lesion) showing significant reduction in the median K^trans^(min^−1^) with accompanying histogram (whole lesion) at day 15 post-treatment. (B) Coronal CT images at baseline, best response (2 months) and at the end of treatment (14 months) for same liver lesion (left) and an abdo-pelvic mass (right). Patient achieved stable disease by RECIST V.1.1. (C) Matched IHC analysis demonstrating decrease and subsequent increase in tumour vascularity measured by staining CD31 at 2 and 14 months, respectively. (D) Coronal DCE-MRI and accompanying histogram of the liver lesion showing no significant reduction in the median K^trans^(min^−1^) at day 15 post-treatment. (E) Coronal CT images of the liver showing progression (30% increase) of the same target liver lesion (yellow circle) at baseline and at progression (2-month scan). (F) Matched IHC analysis demonstrating no change in tumour vascularity measured by staining CD31 at 2 months. Two separate progressive disease lesions were analysed to take into account tumour heterogeneity; however, no change in vascularity was observed in either of the biopsied lesion. CT, computed tomography; DCE, dynamic contrast enhanced; IHC, immunohistochemistry.

A decrease in CD31 score at 2 months was associated with higher DCR (OR 30.0 (95% CI 2.22 to 405.98), p=0.01), better PFS (HR 0.13 (95% CI 0.03 to 0.52), p=0.004) and better OS (HR 0.30 (95% CI 0.08 to 1.06), p=0.06) (see online appendix figure A2). Examples of KEF drop, RECIST V. 1.1 response and CD31 scoring at different time points in a responder (figure 3A-C) and non-responder patient (figure 3D-F) are provided.

### Radiological and pathological analysis of proliferation and apoptosis following regorafenib treatment

Radiological cell kill effects of regorafenib were investigated by examining the changes in ADC on DW-MRI, pretreatment and at day 15. Matching tissue was scored for cell proliferation (KI-67 index) and apoptosis (caspase 3) at pretreatment and 2 months post-therapy. Median ADC changes are described in online appendix table A7. The changes at 2 months in corresponding tissue parameters of cell proliferation were not associated with an improvement in DCR (OR 1.13 (95% CI 0.14 to 9.0), p=0.91), PFS (HR 1.11 (95% CI 0.35 to 3.58), p=0.86) or OS (HR 0.91 (95% CI 0.19 to 4.42), p=0.91); similarly, no significant changes in apoptosis were observed when comparing baseline and 2 months treatment tissue biopsies.

### Liquid biopsy as a surrogate marker of response to regorafenib

**Figure 4:**
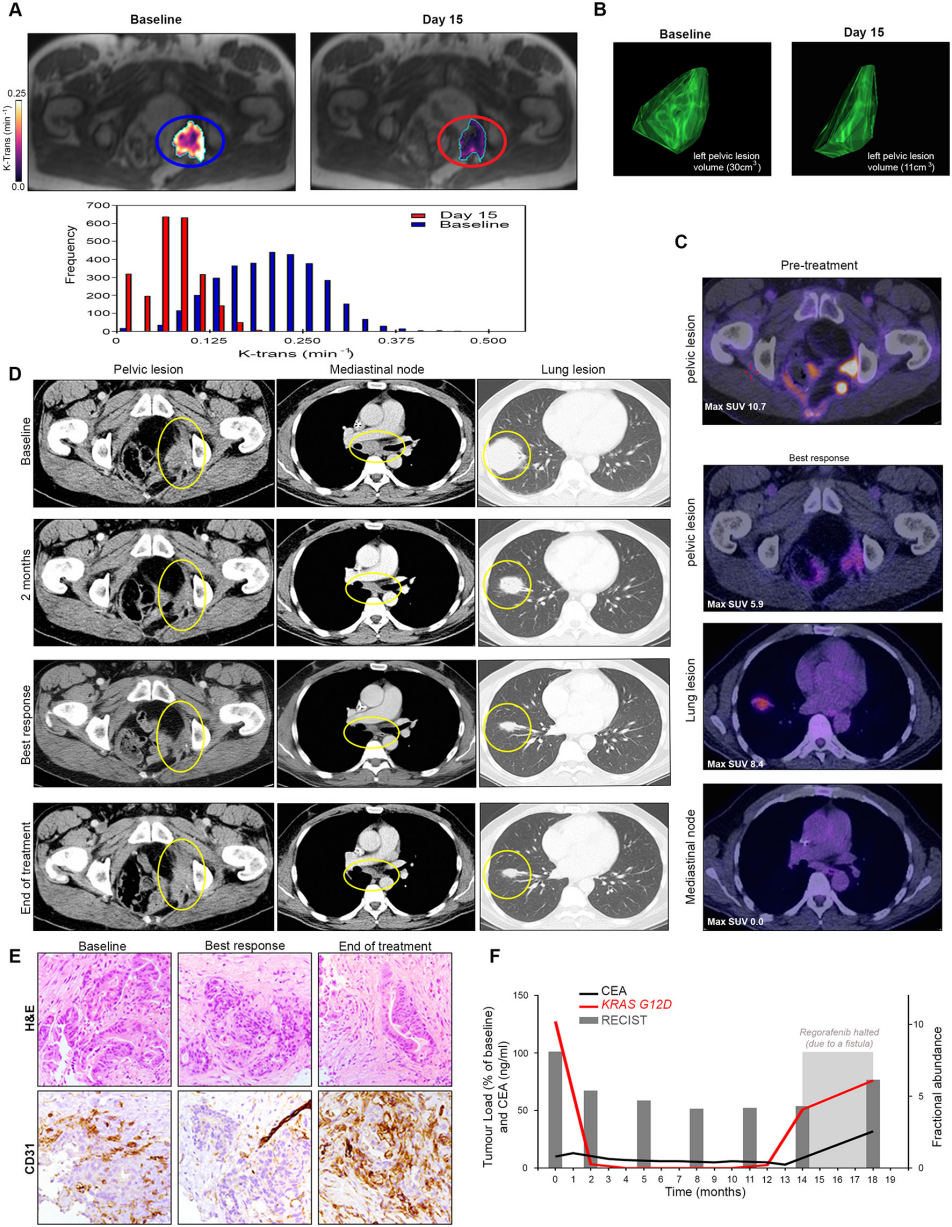
Correlation between radiological, pathological and circulating biomarkers in PROSPECT-R trial. (A) Axial DCE-MRI demonstrating significant reduction (71%) of the median K^trans^(min^−1^) in the left pelvic wall recurrence, with accompanying histogram at day 15 post-regorafenib. (B) Three-dimensional representation of target lesion by CT performed at baseline and at week 31 (best response) demonstrating reduction in lesion volume. (C) FDG-PET images performed at 4 months of therapy showing residual FDG uptake, although significantly less when compared with a historic PET-CT performed 18 months prior to regorafenib therapy. (D) Axial CT images demonstrating a maintained RECIST V.1.1 partial response (45%) to regorafenib for 31 weeks. Images show representative sites of disease including left pelvis side wall, mediastinal lymphadenopathy and large lung metastases (yellow circles). Note is made that at the time of progression, left pelvic side wall disease progressed (28%), while the remaining disease had maintained partial response demonstrating the intertumoural heterogeneity in resistance to regorafenib. (E) Matched IHC analysis demonstrating decrease and subsequent increase in tumour vascularity measured by staining CD31 at 2 and 12 months, respectively. (F) Graphical representation of clonal *KRAS* mutation tracked by digital droplet PCR analysis of circulating tumour DNA analysis compared with CEA and total volume of target lesions measured RECIST V. 1.1 assessment. This demonstrates that an early drop and rise in fractional abundance of *KRAS* mutation that precedes changes in CEA, both at response and resistance to regorafenib. CEA, carcinoembryonic antigen; DCE, dynamic contrast enhanced; FDG-PET, 18 Fluoro-deoxyglucose positron emission tomography; IHC, immunohistochemistry; MRI, magnetic resonance imaging.

We hypothesised that regorafenib-induced anti-angiogenic effects would correlate with a reduction in circulating tumour DNA (ctDNA). Indeed, in a patient with significant (71%) KEF drop after 2 weeks of treatment (figure 4A) and durable RECIST V. 1.1. response lasting nearly 12 months (figure 4B-D), we observed that the KEF reduction was correlated with CD31 drop (figure 4E) and associated with a rapid and marked decrease in *KRAS G12D* ctDNA which persisted for the entire duration of the treatment and increased again when the treatment was halted due to a complication (figure 4F). Intriguingly, the changes in carcinoembryonic antigen lagged behind the changes in ctDNA.

**Figure 5:**
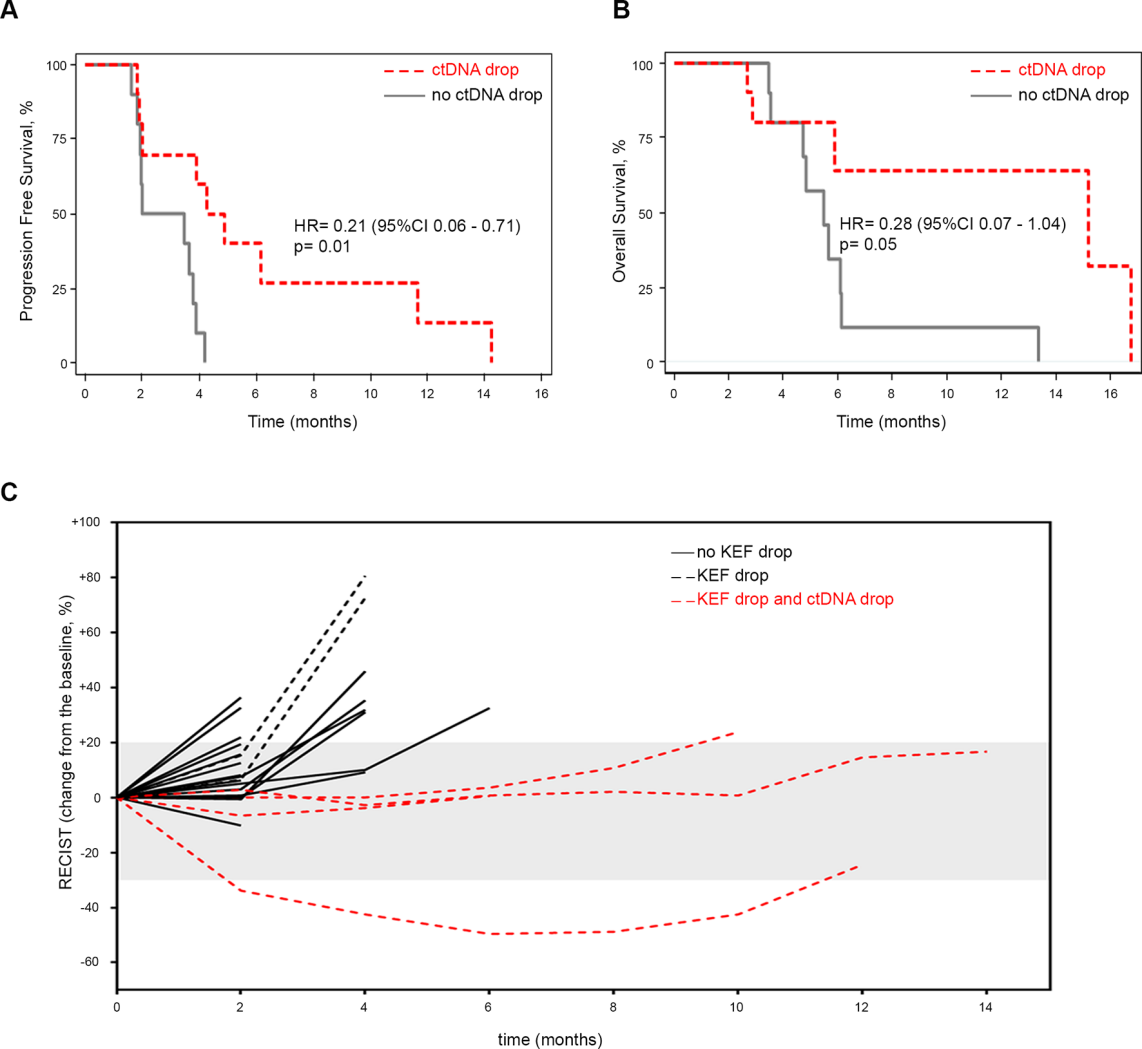
Outcome according to ctDNA drop after 2 months of treatment in the PROPSECT-R trial. Kaplan-Meier curves for progression-free survival (A) and overall survival (B) in patients with or without ctDNA drop, (C) spider plot demonstrating depth and duration of response to regorafenib (evaluated by RECIST V.1.1. criteria) according to KEF and ctDNA drop. ctDNA, circulating tumour DNA; KEF, K^trans^× enhancing fraction.

To test this hypothesis, we analysed changes in *RAS* mutant clones in sequential liquid biopsies by ddPCR. We examined whether a drop in FA was associated with clinical efficacy parameters. We found that the loss of detectable mutant *RAS* clones in ctDNA after 4 weeks was universal to all the examined patients ((n=21) data not shown). However, a sustained drop in ctDNA was observed in 47.6% of the patients at 2 months and was associated with better PFS (HR 0.21 (95% CI 0.06 to 0.71), p=0.01) and OS (HR 0.28 (95% CI O.07 to 1.04), p=0.06), respectively (figure 5A,B); PFS was 60.0% (after 4 months) and 40.0% (after 6 months) in the groups with decrease in FA. In a multivariate analysis adjusting for KEF reduction, this effect was associated with better PFS (HR 0.23 (95% CI 0.07 to 0.75), p=0.02).

Despite the small numbers, which precluded any statistical analysis, it was remarkable to observe that patients with a KEF drop >70% and decrease in ctDNA FA had the most durable response to regorafenib (figure 5C).

### Known biomarkers of benefit from regorafenib, toxicity profile and clinical outcome in the PROSPECT-R trial

A previously well-conducted study comprising 208 regorafenib-treated patients demonstrated an association between high neutrophil, high platelet, low lymphocyte count and/or high neutrophil–lymphocyte ratio with prognosis.^26^ Due to the stringent inclusion criteria of our study, our data distribution did not allow to use the same cut-off used in the study by Del Prete and colleagues and median values were used instead. Notwithstanding small numbers and patient selection based on trial inclusion/exclusion criteria, no significant correlation with efficacy was found with any of the above-mentioned factors (see online appendix tables A8 and A9).

Moreover, other clinical factors such as PS and number of previous lines of treatment and toxicity were also compared with efficacy in a univariate analysis. Treatment-related adverse events were consistent with previously reported data^4^ and are summarised in online appendix tables A10 and A11. As expected, patients who required >50% dose reduction and those who received less than two cycles of regorafenib derived less benefit from the treatment (see online appendix tables A12).

## Discussion

This proof-of-concept phase II translational research study was designed to assess the feasibility of combining imaging, morphological and plasma biomarkers in order to best stratify patients more likely to derive benefit from regorafenib in refractory mCRC. Our study provides the first clinical evidence that regorafenib efficacy is driven by its early anti-angiogenic activity.

It is widely accepted that DCE-MRI can assess tumour vascular function^27^; however, establishing common methodology remains challenging due to the practicalities of technical implementation across different MR platforms and the choice of mathematical models for data analysis. In this study, we have used DCE-MRI acquisition and data analysis in line with international expert recommendations.^27^ While a large body of evidence supports the notion that perfusion MRI can be helpful in assisting dose selection and enriching patient populations more likely to respond in early-phase clinical trials, most studies have defined an observable anti-angiogenic drug effect based only on the limits of DCE-MRI measurement repeatability rather than also considering the clinical efficacy.^28^ Furthermore, as metastases show variable degrees of necrosis and non-enhancement before treatment and drug-induced vascular pruning also leads to marked decrease in enhancement within tumours, measuring only the median K^trans^ value is less sensitive to change due to averaging of the voxel values. For these reasons, we calculated the EF and the product of K^trans^ from the enhancing voxels with EF (KEF), which better reflects proportional reduction of vascularity within tumours.^24^

In this study, we have evaluated DCE-MRI in a well-defined study population, thus minimising the bias that may result from patient heterogeneity. The selected DCE-MRI parameter threshold applied for patient stratification is based on both a prior knowledge of the measurement repeatability of our technique^29^ and clinically validated endpoints of PFS and OS. To our knowledge, this is the first prospective study showing that KEF, a product of K^trans^ and EF, can be used as a parameter of DCE-MRI with high clinical specificity. The KEF measurement was able to identify clinically meaningful responders as early as 2 weeks into treatment with regorafenib with 100% specificity.

The major strength of this study is that it was possible to validate the findings of MRI detected regorafenib-induced suppression of tumour vascularisation by matched tissue analysis using immunostaining of the endothelial marker CD31. We demonstrated that patients with a significant drop in CD31 score on 2-month biopsy had a better PFS and OS. These findings further emphasise the fact that drug activity is due to its anti-angiogenic properties.

It is established that genetic and non-genetic mechanisms of tumour heterogeneity allow functional expansion of previously dormant subclones under the selective pressure of chemotherapy in CRC cells.^30^ This provides a strong biological rationale for the use of regorafenib given its broad multikinase antitumour activity. However, the diversity of mechanisms of action of this drug makes it equally challenging to identify predictive biomarkers of clinical utility. Biomarker analysis of CORRECT trial data demonstrated that benefit from regorafenib was independent of the *RAS* pathway mutational status of the tumour, suggesting primarily an anti-angiogenic mechanism of action, and that liquid biopsy could be reliably used to characterise clonal mutations.^8^ We investigated if the circulating tumour genotype could be used as a biomarker of sustained anti-angiogenic activity to regorafenib by tracking known *KRAS* clonal mutations and performing serial plasma analysis by highly sensitive ddPCR methodology, at clinically relevant time points. A drop in FA was observed in all patients at 4 weeks suggesting a degree of initial anti-angiogenic activity in keeping with an initial drop in radiological parameters; however, this effect was sustained in only a proportion of patients at 2 months. This group of patients with persistent drop at 2 months demonstrated better efficacy with regorafenib suggesting that sustained angiogenic activity was required in order to achieve maintained benefit from therapy. Consistent with the findings from previous studies,^25 31^ we demonstrated that ctDNA can be used for tumour genotyping, but beyond this we proved that it can also be used to monitor efficacy from regorafenib in patients showing initial benefit from the therapy.

Acknowledging the limitations due to small numbers of patients in our study, we propose that these findings should be validated in larger cohort of patients treated with anti-angiogenic therapies. Due to logistical barriers, it may however not be possible to conduct large-scale trials scrupulously designed and statistically powered to address questions of biomarker analysis. The interpretation of our findings thus need to be contextualised; for example, regorafenib is currently unavailable free of charge to patients in the UK so the use of biomarkers described in this study could significantly reduce the duration of therapy in patients’ unlikely to derive benefit. It is conceivable that the health economic assessment might be more favourable with appropriate predictive biomarkers such as those we have identified. While the search for a positive predictive biomarker may help better application of precision medicine, in a more non-resource-constrained funding environment, based on our findings, patients could be spared from significant drug-related side effects, which again would have health economic benefits.

In summary, the depth of angiogenic response measured by DCE-MRI and validated by matched tissue immunohistochemistry analysis correlates with clinical efficacy. The circulating tumour genotype is a potential marker of sustained anti-angiogenic response to regorafenib in patients with known clonal mutations.

## References

[1] DeSantis CE, Lin CC, Mariotto AB, et al Cancer treatment and survivorship statistics. CA: A Cancer Journal for Clinicians 2014;64:252–71. 10.3322/caac.21235 24890451

[2] Cancer Genome Atlas Network. Comprehensive molecular characterization of human colon and rectal cancer. Nature 2012;487:330–7. 10.1038/nature11252 22810696 PMC3401966

[3] Bhargava P, Robinson MO Development of second-generation VEGFR tyrosine kinase inhibitors: current status. Curr Oncol Rep 2011;13:103–11. 10.1007/s11912-011-0154-3 21318618 PMC3047052

[4] Grothey A, Van Cutsem E, Sobrero A, et al CORRECT Study Group. Regorafenib monotherapy for previously treated metastatic colorectal cancer (CORRECT): an international, multicentre, randomised, placebo-controlled, phase 3 trial. Lancet 2013;381:303–12. 10.1016/S0140-6736(12)61900-X 23177514

[5] Li J, Qin S, Xu R, et al CONCUR Investigators. Regorafenib plus best supportive care versus placebo plus best supportive care in Asian patients with previously treated metastatic colorectal cancer (CONCUR): a randomised, double-blind, placebo-controlled, phase 3 trial. Lancet Oncol 2015;16:619–29. 10.1016/S1470-2045(15)70156-7 25981818

[6] Guinney J, Dienstmann R, Wang X, et al The consensus molecular subtypes of colorectal cancer. Nat Med 2015;21:1350–6. 10.1038/nm.3967 26457759 PMC4636487

[7] Gourley CMA, Perren T, Paul J, et al Molecular subgroup of high-grade serous ovarian cancer (HGSOC) as a predictor of outcome following bevacizumab, 2014.

[8] Tabernero J, Lenz HJ, Siena S, et al Analysis of circulating DNA and protein biomarkers to predict the clinical activity of regorafenib and assess prognosis in patients with metastatic colorectal cancer: a retrospective, exploratory analysis of the CORRECT trial. Lancet Oncol 2015;16:937–48. 10.1016/S1470-2045(15)00138-2 26184520 PMC7513622

[9] Giampieri R, Salvatore L, Del Prete M, et al Angiogenesis genotyping and clinical outcome during regorafenib treatment in metastatic colorectal cancer patients. Sci Rep 2016;6:25195 10.1038/srep25195 27117754 PMC4846860

[10] Suenaga M, Mashima T, Kawata N, et al Serum VEGF-A and CCL5 levels as candidate biomarkers for efficacy and toxicity of regorafenib in patients with metastatic colorectal Cancer. Oncotarget 2016;7:34811-23 doi:10.18632/oncotarget.9187 27166185 10.18632/oncotarget.9187 PMC5085191

[11] Abou-Elkacem L, Arns S, Brix G, et al Regorafenib inhibits growth, angiogenesis, and metastasis in a highly aggressive, orthotopic colon cancer model. Mol Cancer Ther 2013;12:1322–31. 10.1158/1535-7163.MCT-12-1162 23619301

[12] Wilhelm SM, Dumas J, Adnane L, et al Regorafenib (BAY 73-4506): a new oral multikinase inhibitor of angiogenic, stromal and oncogenic receptor tyrosine kinases with potent preclinical antitumor activity. Int J Cancer 2011;129:245–55. 10.1002/ijc.25864 21170960

[13] Fan LC, Teng HW, Shiau CW, et al SHP-1 is a target of regorafenib in colorectal cancer. Oncotarget 2014;5:6243–51. doi:10.18632/oncotarget.2191 25071018 10.18632/oncotarget.2191 PMC4171626

[14] Cyran CC, Kazmierczak PM, Hirner H, et al Regorafenib effects on human Colon carcinoma xenografts monitored by dynamic contrast-enhanced computed tomography with immunohistochemical validation. PLoS One 2013;8:e76009 10.1371/journal.pone.0076009 24098755 PMC3786893

[15] Jackson A, O’Connor JP, Parker GJ, et al Imaging tumor vascular heterogeneity and angiogenesis using dynamic contrast-enhanced magnetic resonance imaging. Clin Cancer Res 2007;13:3449–59. 10.1158/1078-0432.CCR-07-0238 17575207

[16] Morgan B, Thomas AL, Drevs J, et al Dynamic contrast-enhanced magnetic resonance imaging as a biomarker for the pharmacological response of PTK787/ZK 222584, an inhibitor of the vascular endothelial growth factor receptor tyrosine kinases, in patients with advanced colorectal cancer and liver metastases: results from two phase I studies. J Clin Oncol 2003;21:3955–64. 10.1200/JCO.2003.08.092 14517187

[17] Tuncbilek N, Karakas HM, Altaner S Dynamic MRI in indirect estimation of microvessel density, histologic grade, and prognosis in colorectal adenocarcinomas. Abdom Imaging 2004;29:166–72. 10.1007/s00261-003-0090-2 15290941

[18] Padhani AR, Liu G, Koh DM, et al Diffusion-weighted magnetic resonance imaging as a cancer biomarker: consensus and recommendations. Neoplasia 2009;11:102–25. 10.1593/neo.81328 19186405 PMC2631136

[19] Li SP, Padhani AR Tumor response assessments with diffusion and perfusion MRI. J Magn Reson Imaging 2012;35:745–63. 10.1002/jmri.22838 22434697

[20] Kazmierczak PM, Burian E, Eschbach R, et al Monitoring Cell Death in Regorafenib-Treated Experimental Colon Carcinomas Using Annexin-Based Optical Fluorescence Imaging Validated by Perfusion MRI. PLoS One 2015;10:e0138452 10.1371/journal.pone.0138452 26393949 PMC4578959

[21] d’Arcy JA, Collins DJ, Padhani AR, et al Informatics in Radiology (infoRAD): Magnetic Resonance Imaging Workbench: analysis and visualization of dynamic contrast-enhanced MR imaging data. Radiographics 2006;26:621–32. 10.1148/rg.262045187 16549620

[22] Orton MR, d’Arcy JA, Walker-Samuel S, et al Computationally efficient vascular input function models for quantitative kinetic modelling using DCE-MRI. Phys Med Biol 2008;53:1225–39. 10.1088/0031-9155/53/5/005 18296759

[23] Parker GJ, Roberts C, Macdonald A, et al Experimentally-derived functional form for a population-averaged high-temporal-resolution arterial input function for dynamic contrast-enhanced MRI. Magn Reson Med 2006;56:993–1000. 10.1002/mrm.21066 17036301

[24] Ferl GZ, O’Connor JP, Parker GJ, et al Mixed-effects modeling of clinical DCE-MRI data: application to colorectal liver metastases treated with bevacizumab. J Magn Reson Imaging 2015;41:132–41. 10.1002/jmri.24514 24753433

[25] Siravegna G, Mussolin B, Buscarino M, et al Clonal evolution and resistance to EGFR blockade in the blood of colorectal Cancer patients. Nat Med 2015;21:827 10.1038/nm0715-827b 26151329

[26] Del Prete M, Giampieri R, Loupakis F, et al Prognostic clinical factors in pretreated colorectal Cancer patients receiving regorafenib: implications for clinical management. Oncotarget 2015;6:591–2. 10.1200/jco.2015.33.3_suppl.591 PMC4741819 26334693

[27] Leach MO, Morgan B, Tofts PS, et al Imaging vascular function for early stage clinical trials using dynamic contrast-enhanced magnetic resonance imaging. Eur Radiol 2012;22:1451–64. 10.1007/s00330-012-2446-x 22562143

[28] Zweifel M, Padhani AR Perfusion MRI in the early clinical development of antivascular drugs: decorations or decision making tools? Eur J Nucl Med Mol Imaging 2010;37(S1):164–82. 10.1007/s00259-010-1451-z 20461374

[29] Rata M, Collins DJ, Darcy J, et al Assessment of repeatability and treatment response in early phase clinical trials using DCE-MRI: comparison of parametric analysis using MR- and CT-derived arterial input functions. Eur Radiol 2016;26:1991–8. 10.1007/s00330-015-4012-9 26385804 PMC4902841

[30] Kreso A, O’Brien CA, van Galen P, et al Variable clonal repopulation dynamics influence chemotherapy response in colorectal Cancer. Science 2013;339:543–8. 10.1126/science.1227670 23239622 PMC9747244

[31] Bettegowda C, Sausen M, Leary RJ, et al Detection of circulating tumor DNA in early- and late-stage human malignancies. Sci Transl Med 2014;6:224ra24 10.1126/scitranslmed.3007094 PMC4017867 24553385

