# Supplementary material for: Functional imaging and circulating biomarkers of response to regorafenib in treatment-refractory metastatic colorectal cancer patients in a prospective phase II study
Source: Gut. 2017 Aug 8;67(8):1484–92. doi: 10.1136/gutjnl-2017-314178 (PMC6204951; doi:10.1136/gutjnl-2017-314178)
Supplement: Supplementary Appendix 1 [file gutjnl-2017-314178supp001.pdf]

Appendix to: Functional Imaging and circulating biomarkers of response to Regorafenib in treatment-refractory metastatic colorectal cancer patients in a PROSPECTive phase II study.

Authors: Khurum Khan, Mihaela Rata, David Cunningham', Dow-Mu Koh, Nina Tunariu, Jens C Hahne, George Vlachogiannis, Somaieh Hedayat, Silvia Marchetti, Andrea Lampis, Mahnaz Darvish Damavandi, Hazel Lote, Isma Rana, Anja Williams, Suzanne A Eccles, Elisa Fontana, David Collins, Zakaria Eltahir, Sheela Rao, David Watkins, Naureen Starling, Jan Thomas, Eleftheria Kalaitzaki, Nicos Fotiadis, Ruwaida Begum, Maria Bali, Massimo Rugge, Eleanor Temple, Matteo Fassan, Ian Chau, Chiara Braconi, & Nicola Valeri

INDEX OF SUPPLEMENTARY MATERIAL:

Methods 2

Supplementary Tables

Appendix Table A1: Concordance MRI-Biopsy 11
Appendix Table A2: Summary of changes in DCE-MRI parameters 12
Appendix Table A3: ROC curve analysis to choose clinically appropriate KEF drop 13
Appendix Table A4: Correlation between KEF and CD31 drop 14
Appendix Table A5: (K^{trans} * EF/100) according to RECIST response (70%) 15
Appendix Table A6: (K^{trans} * EF) and correlation with clinical efficacy parameter 16
Appendix Table A7: Apparent Diffusion Coefficient (ADC) changes on day 15 17
Appendix Table A8: Association of PFS with clinical/haematological factors 18
Appendix Table A9: Association of OS with clinical/haematological factors 19
Appendix Table A10: Summary of dose adjustments required on the study 20
Appendix Table A11: Summary of grade 3-5 toxicities on the study 22
Appendix Table A12: Dose adjustments and outcomes in pts with >70% KEF drop 23

Supplementary Figures

Appendix Figure A1: Waterfall plot representing KEF drop after 15 days of treatment 24
Appendix Figure A2: Outcome according to CD-31 drop after 2 months of treatment 25

References 26

Methods

Study inclusion criteria (as specified in trial protocol):

1. In order to be eligible for registration, all inclusion criteria must be met. A patient must:
   - Understand, be willing to give consent, and sign the written informed consent form (ICF) prior to undergoing any study-specific procedure:
   - Be male or female and ≥ 18 years of age
2. patients with a histologically confirmed diagnosis of metastatic colorectal adenocarcinoma, have RAS MT disease (Patients who are undergoing biopsies for diagnostic purposes will be allowed to participate in the study, as long as the diagnostic test confirms the evidence of RAS mutant disease) and have received the following treatment regimens described below: Previous treatment with fluoropyrimidine-,oxaliplatin-, and irinotecan-based chemotherapy; and progressed following the last administration of approved therapy. Subjects who have discontinued treatment due to unacceptable toxicity will also be allowed into the study.

3. patients with inoperable mCRC who are suitable for treatment with regorafenib as monotherapy and had: a CT or MRI scan (chest, abdomen, pelvis and other suspected sites as applicable) to determine eligibility for recruitment within 4 weeks prior to treatment (hereafter referred to as the "Eligibility scan")
4. patients who have metastatic disease sites which are amenable to core biopsy (preferably liver, soft tissue or nodal disease, with at least one lesion 1.5cm or more in diameter. If largest lesion 1-1.5cm diameter, eligibility to be discussed with radiologist prior to study entry)
5. patients who have an Eastern Cooperative Oncology Group (ECOG) performance status of 0 or 1 within 28 days prior to the initiation of study treatment
6. patients who have adequate bone marrow, liver function, and renal function, as measured by the following laboratory assessments conducted within 14 days prior to the initiation of study treatment:

- Total bilirubin < 1.5 times the upper limit of normal (ULN)
- Alanine aminotransferase (ALT) and aspartate aminotransferase (AST) < 2.5 times the ULN in patients with no hepatic metastases and <5 the ULN in patients with hepatic metastatic disease.
- Glomerular filtration rate (GFR) ≥ 30 mL/min/1.73 m2 according to the modified diet in renal disease (MDRD) abbreviated formula.
- Platelet count 100000 /mm3, hemoglobin (Hb) 9 g/dL, absolute neutrophil count (ANC) ^{3} 1500/mm3
- Lipase < 1.5 x ULN
- International normalized ratio (INR) of prothrombin time (PT; PT-INR) and partial thromboplastin time (PTT) ≤ 1.5 times the ULN. Patients who are therapeutically treated with an agent such as warfarin or heparin will be allowed to participate if no underlying abnormality in coagulation parameters exists as per medical history. Weekly evaluation of PT-INR/PTT will be required until stability is achieved (as defined by local standard of care and based on pre-study PT-INR/PTT values). The anti-coagulation therapy will be stopped 48 hours prior to the biopsy and re-commenced 24-48 hours after the procedure on recommendation of the interventional radiologist. Physicians will be strongly encouraged to switch oral coumadin derivatives (e.g. warfarin) to subcutaneous formulations, however if this was not possible due to any clinical reasons or patients preference, they will still be allowed on the study with careful monitoring of their INR and after discussion with the interventional radiologist performing the procedure.

7. If female and of childbearing potential, have a NEGATIVE result on a pregnancy test performed a maximum of 7 days before initiation of study treatment; pregnancy status must be documented prior to the first dose of study treatment

8. If female and of childbearing potential or if male, must agree to use adequate contraception (e.g., abstinence, intrauterine device, oral contraceptive, or double-barrier method) based on the judgment of the investigator or a designated associate from the date on which the ICF is signed until 6 months after the last dose of study drug.

Study exclusion criteria **(as specified in trial protocol)**

A patient who meets **ANY** of the exclusion criteria will **NOT** be eligible for randomization.

A patient must **NOT**

1. have had prior treatment with regorafenib or any other VEGF-targeting kinase inhibitor
2. have had previous or concurrent cancer that is distinct in primary site or histology from colorectal cancer within 2 years prior to recruitment EXCEPT for curatively treated cervical cancer in situ, non-melanoma skin cancer and superficial bladder tumors [Ta (Noninvasive tumor), Tis (Carcinoma in situ) and T1 (Tumor invades lamina propria)].
3. Patients that are participating in another clinical trial involving an investigational medicinal product, unless it is more than 14 days after they have ceased the investigational medicinal product
4. Patients that are participating in another research study involving tumour tissue biopsies planned to take place during the time that the patient is participating in this study
5. Have had a major surgical procedure, open biopsy, or significant traumatic injury within 28 days prior to initiation of study treatment
6. If female and of childbearing potential, be engaged in breast feeding
7. Be unable to swallow oral tablets (crushing of study treatment tablets is not allowed)
8. Arterial or venous thrombotic or embolic events such as cerebrovascular accident (including transient ischemic attacks), deep vein thrombosis or pulmonary embolism within 6 month before the start of study medication (except for adequately treated

catheter-related venous thrombosis occurring more than one month before the start of study medication)

9. Interstitial lung disease with ongoing signs and symptoms at the time of informed consent.
10. Ongoing infection > Grade 2 NCI CTCAE
11. Uncontrolled hypertension (Systolic blood pressure > 140 mmHg or diastolic pressure > 90 mmHg) despite optimal medical management
12. Have congestive heart failure classified as New York Heart Association Class 2 or higher
13. Have had unstable angina (angina symptoms at rest) or new-onset angina < 3 months prior to screening
14. Have had a myocardial infarction < 6 months prior to initiation of study treatment
15. Have cardiac arrhythmias requiring anti-arrhythmic therapy, with the exception of beta blockers, calcium channel blockers or digoxin
16. Have pheochromocytoma
17. Have a known history of human immunodeficiency virus infection
18. Have either active hepatitis B or C or chronic hepatitis B or C requiring treatment with antiviral therapy
19. have an active unstable seizure disorder with last episode of seizure within 4 weeks of starting the trial treatment
20. Have had a hemorrhage or a bleeding event Grade 3 (NCI-CTCAE v 4.0) within 4 weeks prior to the initiation of study treatment
21. Have a non-healing wound, ulcer, or bone fracture
22. Have renal failure requiring hemodialysis or peritoneal dialysis
23. Have persistent proteinuria > 3.5 g/24 h, measured by urine protein:creatinine ratio from a random urine sample (Grade 3, NCI-CTCAE v 4.0)
24. Have a substance abuse, medical, psychological, or social condition that may interfere with participation in the study or evaluation of the study results

25. Have a known hypersensitivity to any of the study drugs, study drug classes, or excipients in the formulation of the study drugs
26. Have history of brain metastases

Subject withdrawal criteria **(as specified in trial protocol)**

Participation in this study is voluntary. Patients will be permitted to withdraw from the study at any time.

If a patient's scheduled dose of regorafenib treatment is delayed for more than 4 weeks for any reason, or if a patient ceases treatment because of toxicity, then the patient will withdraw from this study and will not be asked to undergo the second mandatory or the optional research biopsy, unless the patient has clinically and/or radiologically progressed.

If a patient is not able to have a biopsy because they are no longer considered fit for biopsy, or there is no longer a site suitable for biopsy, then the patient will not be required to undergo further biopsies. This will need to be discussed with the Chief Investigator. These patients will be required to be replaced to complete 30 evaluable patients with paired biopsies; however, the clinical and translational data generated from the withdrawn patients will be reported as part of the study.

If a patient loses capacity to consent during the study, then the patient would withdraw from the study.

Should a patient withdraw from the study, then any biological material and data collected during the study period may still be analysed, unless the patient specifically requests that this does not occur. If the patient consents, serious adverse event data will continue to be collected for 30 days after the last procedure, even if the patient has withdrawn from the

study. Despite treatment withdrawal, patients will continue to be followed in the study. The frequency of follow-up is left to the clinician’s discretion.

However, survival status should be ascertained at least once every 3 months which can be conducted over the telephone alone.

MRI data acquisition

All patients were scanned on a MAGNETOM Avanto 1.5T MR scanner (Siemens Healthcare, Erlangen, Germany) Before any post-processing, DCE-MRI liver data were manually registered (2D technique, in house software) to minimise any residual breathing effect. Regions of interest (ROIs) of the imaged target lesion were drawn, slice by slice, by a senior radiologist on high-b-value diffusion weighted images (b900) and translated to the DCE-MRI data. Voxel-wise analysis of the delineated ROIs was performed using in-house written software designed for each imaging technique [1]. For all imaging parameters, the results of each analysed image section were merged to obtain a volume of interest (VOI); the number of image sections (2-10) included in the VOI depended on the lesion size; the median value of VOI imaging parameters for every patient at each time point was reported. The ADC was calculated assuming a mono-exponential fitting algorithm.

DCE protocol: A standard dose of contrast agent (Dotarem, 0.2 ml/kg) followed by 20 ml of saline was delivered by an automatic power injector at 3 ml/s. DCE-MRI data were acquired using a 3D fast field echo sequence with: 14 coronal partitions, slice thickness 5mm, TR/TE = 3/0.89 ms, flip angle = 11°, FOV=400x400 mm^2, matrix=128x128, 1 average, parallel acquisition (Grappa acc. factor 2, ref lines 24). Dynamic scans were preceded by a calibration scan with the same parameters, but at a lower flip angle (2°) and with 7 averages, to enable contrast quantification[2]. For abdominal disease sites, patients were imaged coronally using a sequential breath-hold technique optimised for liver lesions: two image volumes were acquired during each 6 s breath-hold, followed by a 6 s breathing gap; 40

volumes were acquired over 4.18 min[3]. For pelvic disease sites, patients were imaged axially with a free breathing technique: 80 image volumes acquired continuously at 3.3 s/vol for 4.4 min.

DWI protocol: Pelvic (axial plane) and abdominal (coronal plane) DWI data were acquired in free breathing. The DWI parameters for liver acquisition were: 2D echo planar imaging sequence, 20 coronal slices, slice thickness 5 mm, TR/TE=5000/60 ms, FOV=400x400 mm^2, 5 independent acquisitions (no averaging), matrix 128x128, phase partial Fourier 7/8, parallel acquisition (GRAPPA acc. factor 2, ref lines 30), 8 b-values (0,20,40,60,120,240,480,900 s/mm^2), diffusion times δ=14.6 ms and Δ=24 ms, total acquisition time ~2 min/acquisition. Similar axial acquisitions were acquired for the pelvic region.

Isolation of circulating tumour (ct)DNA

ctDNA was extracted from EDTA anti-coagulated blood within 1 h after collection, plasma was separated from the cells by centrifugation (1500*g* for 15 min at 4 °C) followed by a second centrifugation of the supernatant at 1500*g* for 10 min at 4 °C to remove all cell debris. If not used immediately, plasma was frozen at -80 °C until further processing. ctDNA from 2 ml of plasma was isolated by the use of Qiagen blood mini kit (Qiagen, Hilden, Germany) according to manufacturer’s protocol.

Statistical Analysis

1) Sample Size (as specified in trial protocol)

Since this study was exploratory in nature and included two (or three) biopsies per patient, we kept the sample size low; to 20 patients with at least two biopsies; i.e. at baseline and at progression. Patients with stable disease or response after 8 weeks were required to have three biopsies. We planed to compare the tumour molecular signatures of patients at commencement of regorafenib with that at the time of progressive disease. It was expected

that 1 to 2 patients would be recruited per month. If any patient refused or withdrew before a second biopsy at the time of disease progression, then that patient was required to be replaced. The sample size was later expanded to 30 patients with paired biopsies through a protocol amendment as half of the patients progressed without any benefit from therapy. Although patients with primary progression would provide valuable information about the reasons behind primary progression but we optimised the sample size in order to have maximum information about the mechanisms of acquired resistance to regorafenib. As indicated in the main body of manuscript, patients meeting the criteria for MRI substudy were included in this cohort analysis.

2) Statistical Analysis of plan on the study (as specified in trial protocol)

The changes in the tumour molecular signature between commencement of regorafenib and development of resistance to the drug will be described at the time of data maturation. Resistance to regorafenib will be defined as the time that the patient ceases regorafenib therapy because of a clinical decision (made by the patient's treating oncologists) to stop treatment due to progressive disease.

Survival endpoints will be analysed using Kaplan Meier methods and median survival presented with 95% confidence intervals. PFS defined as time from start of regorafenib treatment to first progression or death of any cause. OS defined as time from start of regorafenib treatment to death of any cause. Patients who are event free at the time of analysis will be censored.

Candidate genes in circulating free tumour DNA will be tested in the blood samples that are being collected every four weeks. Changes in the candidate genes across time will be described in all patients and also separately for those that achieve disease control and those

that progress (changes up to progression). We will particularly assess whether mutation detection in candidate drivers of regorafenib resistance correlated with primary resistance and whether such candidate resistance drivers become detectable before radiological progression is observed. This may allow the development of minimally invasive therapy stratification biomarkers.

In the patients who have had a tumour biopsy at 8 weeks, the changes in the transcriptomic and genetic signature from baseline to disease control will be described. Disease control rate is defined as partial or complete response or stable disease according to RECIST 1.1.

Objective response rate defined as partial or complete response according to RECIST 1.1 will be summarised as a proportion with 95% confidence interval. Disease control is defined as objective response or stable disease.

Efficacy endpoints (response and survival) will be summarised descriptively by histological growth patterns. Due to small numbers no formal statistical testing will be undertaken.

Changes in the genomic landscape from the time of diagnosis of CRC (using archival tissue) to the biopsy taken before regorafenib treatment and finally until regorafenib resistance has developed will be described. This will provide the first insight into CRC evolution throughout multiple lines of combination chemotherapy, anti-angiogenic treatment and regorafenib therapy. This should provide critical data to define rational re-biopsy strategies throughout CRC patient pathways.

Supplementary Tables

Appendix Table A1: Concordance between DCE-MRI and tissue biopsy. Liver lesions were chosen to optimise the chances of matched tissue analysis as the study involved repeated biopsies and liver is site that can be more conveniently subjected to multiple biopsies. We however didn't find any significant differences in data interpretation depending upon site of metastatic disease. Interestingly, 1 patient with pelvic mass (patient 1), who had >70% drop in KEF was found to have PR with regorafenib. This was the only patient who achieved PR on the current cohort.

Patient ID	Biopsied Target	Biopsy Guidance	Concordance MRI-Tissue Biopsy	Time-lapse between BL MRI and biopsy (less 1 week)
1	left pelvis wall	CT	yes	yes
2	pelvis mass	CT	yes	yes
3	liver: segm 3	US	yes	yes
4	liver: segm 6/7	US	yes	yes
5	pelvic mass	CT	No (MRI of segm8liver)	yes
6	peritoneal	CT/US	No (MRI of segm1liver)	yes
7	liver: segm 5	CT	yes	no (9 days)
8	liver: segm 5	CT/US	yes	yes
9	liver: segm 7	US	yes	yes
10	liver: segm 6	US	yes	yes
11	liver: segm 7	CT	yes	yes
12	liver: segm 5	US	No (MRI of segm8liver)	yes
13	liver: segm 7/8	US	yes	yes
14	liver: segm 8	US	yes	yes
15	liver: segm 3	US	yes	yes
16	liver: segm 6/7	US	yes	yes
17	liver: segm 6	US	yes	yes
18	liver: segm 2/3	US	yes	yes
19	liver: segm 2	US	yes	yes
20	liver: segm 6	US	yes	yes
21	liver: segm 3	US	yes	yes
22	liver: segm 7	US	yes	yes
23	liver: segm 6	US	yes	yes
24	liver: segm 6	US	yes	yes
25	liver: segm 6/7	US	yes	yes
26	liver: segm 6	US	yes	yes
27	liver: segm 7	US	yes	yes

Segm= segment; CT=Computed Tomography; US= Ultrasound; MRI=Magnetic Resonance Imaging; BL=baseline

Appendix Table A2: Summary of changes in DCE-MRI parameters

	K^{trans} (nonzeros) [min^{-1}]	*$IAUGC_{60}$ [mmol·s]*	*EF [%]*	*KEF [min^{-1}]*
**Baseline**				
Mean (sd)	0.14 (0.08)	11.91 (6.01)	88.05 (13.14)	0.13 (0.08)
Median (IQR)	0.11 (0.09 - 0.19)	10.96 (7.07 - 15.26)	92.29 (82.75 - 99.31)	0.11 (0.07 - 0.18)
Range	0.06 - 0.37	2.75-27.87	50.81 - 100.00	0.03 - 0.33
**C1D15**				
Mean (sd)	0.10 (0.07)	5.66 (4.08)	57.86 (23.09)	0.06 (0.05)
Median (IQR)	0.07 (0.07 - 0.10)	5.05 (2.79 - 7.04)	57.94 (41.01 - 77.92)	0.05 (0.03 - 0.07)
Range	0.03 - 0.38	2.04 - 21.97	21.99- 96.87	0.01 - 0.27
**Percentage decrease**				
Mean (sd)	25.20 (34.56)	49.26 (24.58)	34.31 (24.22)	51.29 (27.42)
Median (IQR)	27.75 (6.74 - 52.56)	57.70 (32.66 - 67.93)	35.33 (12.40 - 56.19)	58.28 (28.28 - 76.14)
Range	-63.22 - 82.32	-1.71 - 77.68	-14.03 - 74.76	-5.55 - 93.76

EF= enhancing fraction; IQR= inter-quartile range; sd=standard deviation; C1D15= cycle 1, day 15, KEF= product of summarised median values of K^{trans} (nonzeros) x EF; $IAUGC_{60}$= initial area under the gadolinium concentration-time curve over 60 seconds

Appendix Table A3: ROC curve analysis to choose clinically appropriate KEF drop

Cut point	*Sensitivity*	*Specificity*	*Correctly Classified*
5.55	100.00%	0.00%	60.87%
-4.63	92.86%	0.00%	56.52%
-10.01	85.71%	0.00%	52.17%
-14.38	85.71%	11.11%	56.52%
-17.87	85.71%	22.22%	60.87%
-18.25	78.57%	22.22%	56.52%
-28.27	78.57%	33.33%	60.87%
-32.49	71.43%	33.33%	56.52%
-41.55	71.43%	44.44%	60.87%
-45.47	71.43%	55.56%	65.22%
-51.59	71.43%	66.67%	69.57%
-53.69	64.29%	66.67%	65.22%
-58.28	57.14%	66.67%	60.87%
-65.25	57.14%	77.78%	65.22%
-66.37	50.00%	77.78%	60.67%
-67.12	50.00%	88.89%	65.22%
**-69.21**	**50.00%**	**100.00%**	**69.57%**
-70.99	42.86%	100.00%	65.22%
-77.37	35.71%	100.00%	60.87%
-77.94	28.57%	100.00%	56.52%
-78.86	21.43%	100.00%	52.17%
-82.04	14.29%	100.00%	47.83%
-93.76	7.14%	100.00%	43.48%
-93.76	0.00%	100.00%	39.13%

Appendix Table A4: Correlation between KEF and CD31 drop

	**Drop in KEF**		
**CD31**	No	Yes	Total
No change	7 *(54%)*	0	7 *(37%)*
Drop (i.e. >5% drop from baseline)	6 *(46%)*	6 *(100%)*	12 *(63%)*
Total	13 *(100%)*	6 *(100%)*	19 *(100%)*
	**Fisher's exact, p=0.04**		

Appendix Table A5: KEF (K^{trans} * EF/100) according to RECIST response (70%)

	Responder (CR/PR/SD)		Total
	No (n=9)	Yes (n=14)	(n=23)
No KEF Drop	9	8	17
KEF Drop	0	6	6
Fisher's exact p-value	0.048		
Sensitivity, 95% CI	42.9% (17.7-71.1%)		
Specificity, 95% CI	100% (66.4-100%)		
Accuracy	65.2%		
OR (95% CI)	NE (1.46-NE)		

CI=confidence interval; CR= complete response; n=number; NE= not evaluable; OR= odds ratio; PR= partial response; RECIST=response evaluation criteria in solid tumours; SD= stable disease

Appendix Table A6: KEF (K^{trans} * EF) and correlation with clinical efficacy parameters

	No KEF drop	KEF Drop
**PFS**		
Events	16/17	6/6
Median PFS (95% CI), months	2.0 (1.8-3.9)	5.6 (3.8-NE)
4 months PFS	23.5% (7.3%- 44.9%)	66.7% (19.5% - 90.4%)
6 months PFS	NE	50.0% (11.9% - 80.4%)
HR (95% CI)	*reference*	0.16 (0.04-0.72), p=0.02
**OS**		
Events	12/17	3/6
Median OS (95% CI), months	5.5 (3.4-6.1)	15.2 (6.1-NE)
4 months OS	69.0 (40.8% - 85.5%)	100% (NE)
6 months OS	27.6% (7.2% - 53.2%)	100% (NE)
1 year OS	13.8% (1.0% - 42.5%)	75.0% (12.8% - 96.1%)
HR (95% CI)	*reference*	0.08 (0.01-0.63), p=0.02

CI=confidence interval; HR= hazard ratio; PFS= progression free survival; OS=overall survival

Appendix Table A7: Apparent Diffusion Coefficient (ADC) changes on day 15

Number	*Baseline* [10^{-5} mm^2/s]	*C1D15* [10^{-5} mm^2/s]	*Relative change from baseline* [%]	**Response**
1	101.81	99.03	2.73	0
2	106.83	97.27	8.95	0
3	78.93	100.22	-26.97	0
4	93.77	103.6	-10.48	0
5	108.47	117.04	-7.90	0
6	112.76	114.75	-1.76	0
7	116.47	125.17	-7.47	0
8	85.14	86.24	-1.29	0
9	116.19	119.26	-2.64	0
10	120.72	124.77	-3.35	0
11	112.63	161.71	-43.58	0
12	91.55	103.8	-13.38	0
13	83.82	94.39	-12.61	0
14	118.67	124.74	-5.12	0
15	140.07	150.18	-7.22	0
16	117.57	127.01	-8.03	0
17	98.66	91.16	7.60	0
18	121.47	134.12	-10.41	0
19	124.64	141.39	-13.44	0
20	93.21	118.44	-27.07	0
21	102.08	107.04	-4.86	0
22	102.19	131.36	-28.54	0
23	94.15	111.29	-18.20	0
24	93.5	99.88	-6.82	0
25	95.65	99.01	-3.51	0
26	114.44	124.14	-8.48	0
27	77.73	83.83	-7.85	0

Appendix Table A8: Association of progression free survival with clinical/haematological factors

	Group A	Group B
**PFS in months according to Platelets**		
	**<median (261)**	**≥median (261)**
Events	8/11	7/12
Median PFS (95% CI), months	3.9 (1.8-5.6)	2.0 (1.8 – 4.9)
HR (95% CI)	*reference*	1.02 (0.42-2.48), p=0.953
**PFS in months according to NLR**		
	**<median (4.46)**	**≥median (4.46)**
Events	5/11	10/12
Median PFS (95% CI), months	3.9 (1.8-5.6)	2.0 (1.8 – 4.9)
HR (95% CI)	*reference*	1.50 (0.63-3.59), p=0.364
**PFS in months according to line of treatment**		
	**≤2 lines**	**>2 lines**
Events	7/11	8/12
Median PFS (95% CI), months	1.9 (1.6-3.9)	3.9 (1.9-6.1)
HR (95% CI)	*reference*	0. 43 (0.16-1.11), p=0.80
**PFS in months according to Performance Status**		
	**PS0**	**PS1**
Events	4/7	11/16
Median PFS (95% CI), months	3.6 (1.8-NE)	3.5 (1.8-4.2)
HR (95% CI)	*reference*	2.27 (0.74-694), p=0.150

CI=confidence interval; HR= hazard ratio; PFS= progression free survival; NLR= Neutrophil/Lymphocyte Ratio; PS=Performance Status

Appendix Table A9: Association of overall survival with clinical/haematological factors

	Group A	Group B
**OS in months according to Platelets**		
	**<median (261)**	**≥median (261)**
Events	8/11	7/12
Median OS (95% CI), months	5.8 (4.7-6.1)	13.3 (2.9-NE)
HR (95% CI)	*reference*	0. 72 (0.25-2.12), p=0.554
**OS in months according to NLR**		
	**<median (4.46)**	**≥median (4.46)**
Events	5/11	10/12
Median OS (95% CI), months	6.1 (2.7-NE)	5.7 (3.4-6.1)
HR (95% CI)	*reference*	1.52 (0.50-4.67), p=0.463
**OS in months according to line of treatment**		
	**≤2 lines**	**>2 lines**
Events	7/11	8/12
Median OS (95% CI), months	4.8 (2.7-NE)	6.1 (3.4-NE)
HR (95% CI)	*reference*	0. 44 (0.14-1.33), p=0.146
**OS in months according to Performance Status**		
	**PS=0**	**PS=1**
Events	4/7	11/16
Median OS (95% CI), months	13.3 (2.7-NE)	5.7 (4.7-6.1)
HR (95% CI)	*reference*	2.82 (0.75-10.6), p=0.124

CI=confidence interval; HR= hazard ratio; OS= overall survival; NLR= Neutrophil/Lymphocyte Ratio; PS=Performance Status

Appendix Table A10: Summary of dose adjustments required on the study

Dose reductions*			
**Cycle**	**Yes**	**%**	**Total**
C1D1	0	0.0%	27
C1D15	1	4.0%	25
C2	14	58.3%	24
C3	5	38.5%	13
C4	2	15.4%	13
C5	2	33.3%	6
C6	0	0.0%	4
C7	0	0.0%	2
C8	0	0.0%	2
C9	0	0.0%	2
C10	0	0.0%	2
C11	0	0.0%	1
C12	0	0.0%	1
C13	0	0.0%	1
C14	0	0.0%	1
**Dose Delays†**			
**Cycle**	**Yes**	**%**	**Total**
C1D1	0	0.0%	27
C1D15	0	0.0%	25
C2	4	16.7%	24
C3	6	46.2%	13
C4	1	7.7%	13
C5	0	0.0%	6
C6	1	25.0%	4
C7	0	0.0%	2
C8	0	0.0%	2
C9	0	0.0%	2
C10	0	0.0%	2
C11	0	0.0%	1
C12	0	0.0%	1
C13	0	0.0%	1
C14	0	0.0%	1
**Missed days¥**			
**Cycle**	**Yes**	**%**	**Total**
C1D1	11	40.7%	27
C1D15	10	40.0%	25
C2	7	29.2%	24
C3	5	38.5%	13
C4	4	30.8%	13
C5	2	33.3%	6
C6	2	50.0%	4
C7	0	0.0%	2
C8	0	0.0%	2
C9	0	0.0%	2
C10	0	0.0%	2
C11	0	0.0%	1

C12	1	100.0%	1
C13	1	100.0%	1
C14	0	0.0%	1

*96% and 4% of patients required dose reductions due to non-haematological and haematological toxicities respectively. When dose reduction was required, patients were offered 120mg and 80 mg on first and second dose reductions respectively. Patients came off the study if further dose reduction was required. †58% of patients required dose delays due to non-haematological toxicities and 33% for other logistical reasons. ¥Median days missed on treatment were 6 (4-9) days, mean 7(minimum 1 and maximum 14 days).

Appendix Table A11: Summary of grade 3-5 toxicities on the study

Reported Toxicity	***Grade 3-5***	
	No.	%
Rectal perforation*	1	4
Skin rash (desquamation)	1	4
Anaemia	2	7
Diarrhoea	2	7
Haemorrhage	2	7
Fatigue	4	15
Mucositis	3	11
Hand foot syndrome	6	22
Infection	6	22

*This was the only grade 5 toxicity in the reported cohort

Appendix Table A12: Dose adjustments and efficacy outcomes in patients with >70% KEF drop

	Drop in KEF		
**Any dose reduction ≥50%**	No	Yes	Total
No	14 *(82.3%)*	2 (33.3%)	16 *(69.6%)*
Yes	3 *(17.7%)*	4 *(66.7%)*	7 *(30.4%)*
Total	17 *(100%)*	6 *(100%)*	23 *(100%)* Fisher's exact, **p=0.045**
**Any delay**	No	Yes	Total
No	13 *(76.4%)*	2 (33.3%)	15 *(65.3%)*
Yes	4 *(23.6%)*	4 *(66.7%)*	8 *(34.7%)*
Total	17 *(100%)*	6 *(100%)*	23 *(100%)* Fisher's exact, p=0.131
**Number of cycles >2**	No	Yes	Total
No	10 *(58.8%)*	0 (0%)	10 *(65.3%)*
Yes	7 *(41.2%)*	6 *(100%)*	13 *(34.7%)*
Total	17 *(100%)*	6 *(100%)*	23 *(100%)* Fisher's exact, **p=0.019**

KEF= K^{trans} * EF

Supplementary Figures

Appendix Figure A1: Waterfall plot representing KEF drop after 15 days of treatment in responders and non- responders patients

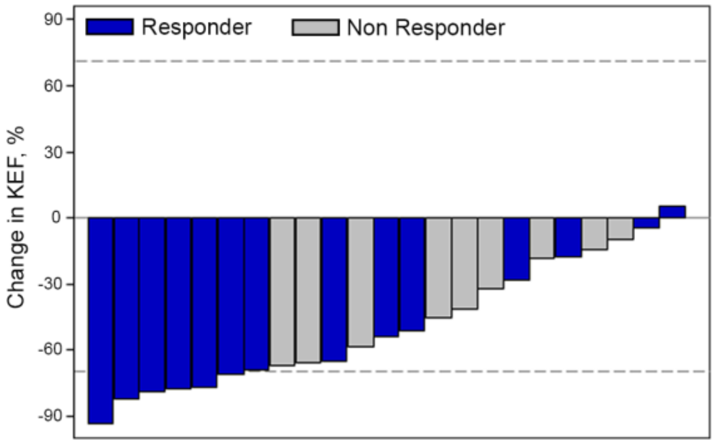

Appendix Figure A1: Waterfall plot of drop vs. no drop in KEF (70%) according to disease control rate measured by RECIST v1.1 at 2 months after initiation of therapy; the blue colour key indicates response (defined as stable disease or partial response by RECIST 1.1) and grey key indicates progressive disease.

Appendix Figure A2: Outcome according to CD-31 drop after 2 months of treatment in the PROSPECT-R Trial

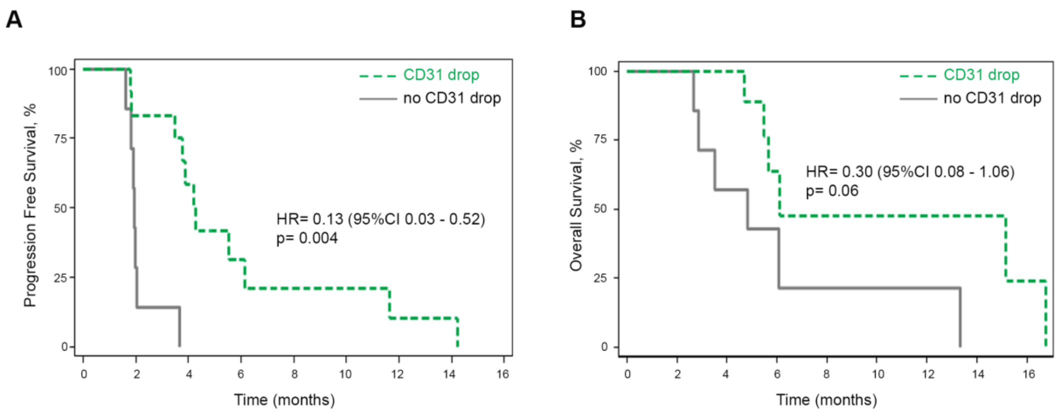

Appendix Figure A2: Kaplan-Meier curves for progression-free survival (**A**) and overall survival (**B**) in patients with or without CD-31 drop.

References

1 d'Arcy JA, Collins DJ, Padhani AR, Walker-Samuel S, Suckling J, Leach MO. Informatics in Radiology (infoRAD): Magnetic Resonance Imaging Workbench: analysis and visualization of dynamic contrast-enhanced MR imaging data. Radiographics : a review publication of the Radiological Society of North America, Inc 2006;**26**:621-32.

2 Fram EK, Herfkens RJ, Johnson GA, Glover GH, Karis JP, Shimakawa A*, et al.* Rapid calculation of T1 using variable flip angle gradient refocused imaging. Magnetic resonance imaging 1987;**5**:201-8.

3 Orton MR, Miyazaki K, Koh DM, Collins DJ, Hawkes DJ, Atkinson D*, et al.* Optimizing functional parameter accuracy for breath-hold DCE-MRI of liver tumours. Physics in medicine and biology 2009;**54**:2197-215.
